# The PATROL1 function in roots contributes to the increase in shoot biomass

**DOI:** 10.1007/s00425-024-04526-8

**Published:** 2024-09-26

**Authors:** Michitaka Notaguchi, Manami Ichita, Takaya Kawasoe, Keina Monda, Ken-ichi Kurotani, Takumi Higaki, Koh Iba, Mimi Hashimoto-Sugimoto

**Affiliations:** 1 Department of Botany, Graduate School of Science, Kyoto University, Kitashirakawa Oiwake-Cho Kyoto, 606–8502 Japan; 2 Bioscience and Biotechnology Center, Nagoya University, Furo-Cho, Chikusa-Ku Nagoya, 464–8601 Japan; 3 Graduate School of Science and Technology, Kumamoto University, Kurokami, Chuo-Ku, Kumamoto, 860–8555 Japan; 4 Department of Biology, Faculty of Science, Kyushu University, Motooka, Nishi-ku Fukuoka, 819–0395 Japan; 5 International Research Organization for Advanced Science and Technology, Kumamoto University, Kurokami, Chuo-Ku, Kumamoto, 860–8555 Japan; 6 International Research Center for Agricultural and Environmental Biology, Kumamoto University, Kurokami, Chuo-Ku, Kumamoto, 860–8555 Japan; 7 Graduate School of Bioagricultural Sciences, Nagoya University, Furo-Cho, Chikusa-Ku, Nagoya, 464–8601 Japan

**Keywords:** Arabidopsis, Biomass, Grafting, H^+^-ATPases, Root meristematic zone, Shoot size

## Abstract

**Main conclusion:**

PATOL1 contributes to increasing biomass not only by effective stomatal movement but also by root meristematic activity.

**Abstract:**

PATROL1 (PROTON ATPase TRANSLOCATION CONTROL 1), a protein with a MUN domain, is involved in the intercellular trafficking of AHA1 H^+^-ATPase to the plasma membrane in guard cells. This allows for larger stomatal opening and more efficient photosynthesis, leading to increased biomass. Although PATROL1 is expressed not only in stomata but also in other tissues of the shoot and root, the role in other tissues than stomata has not been determined yet. Here, we investigated PATROL1 functions in roots using a loss-of-function mutant and an overexpressor. Cytological observations revealed that root meristematic size was significantly smaller in the mutant resulting in the short primary root. Grafting experiments showed that the shoot biomass of the mutant scion was increased when it grafted onto wild-type or overexpressor rootstocks. Conversely, grafting of the overexpressor scion shoot enhanced the growth of the mutant rootstock. The leaf temperatures of the grafted plants were consistent with those of their respective genotypes, indicating cell-autonomous behavior of stomatal movement and independent roles of *PATROL1* in plant growth. Moreover, plasma membrane localization of AHA1 was not altered in root epidermal cells in the *patrol1* mutant implying existence of a different mode of PATROL1 action in roots. Thus PATROL1 plays a role in root meristem and contributes to increase shoot biomass.

**Supplementary Information:**

The online version contains supplementary material available at 10.1007/s00425-024-04526-8.

## Introduction

Plant biomass is largely determined by amount of carbon fixation through stomata gas exchange action. Stomata are small pores in leaf epidermis regulating CO_2_ uptake and transpiration. Stomatal opening is regulated by plasma membrane H^+^-ATPases in response to blue light (Kinoshita et al. 1999, 2001; Kim et al. 2010; Ding et al. 2021). There are 11 isoforms of H^+^-ATPase in Arabidopsis (*Arabidopsis thaliana*), of which the Arabidopsis H^+^-ATPase1, AHA1, is known to be the most important contributor to stomatal movement (Ueno et al. 2005; Yamauchi et al. 2016; Fuglsang and Palmgren 2021). The plasma membrane localization of the AHA1 decreased in stomatal guard cells in *patrol1* (*proton ATPase translocation control 1*) mutant (Hashimoto-Sugimoto et al. 2013). PATROL1 is a protein with a MUN domain, originally found as a large α-helical domain of mammalian Munc13-1. The α-helical domain is the minimal domain responsible for its function, which facilitates formation of soluble N-ethylmaleimide-sensitive factor activating protein receptor (SNARE)-complex required for membrane traffic (Basu et al. 2005). This suggests that PATROL1 localizes AHA1 to the plasma membrane by assisting a fusion of vesicles containing the transporters to the plasma membrane. PATROL1 in guard cells change the localization pattern depending on stomatal opening or closure, and which is opposite in subsidiary cells, indicating the coordinated PATROL1 localization in these cells leads to effective stomatal movements (Higaki et al. 2014). *PATROL1* overexpression line shows a larger seedling phenotype under the short or long day conditions (Hashimoto-Sugimoto et al. 2013), even under fluctuating light (Kimura et al. 2020). Although higher stomatal aperture is expected to allow higher uptake of CO_2_, mutants that fail to close their stomata do not increase biomass due to the loss of excess water (Kimura et al. 2020). The reason for the increased biomass in *PATROL1* overexpressors is that the increased level of H^+^-ATPase in the plasma membrane of guard cells allows stomata to widely open immediately after light exposure, while closing them when necessary, thus leading to effective photosynthesis. Until now, the function of PATROL1 has only been studied in stomatal movement. However, PATROL1 is expressed in most cell types, and their functions except for stomata are unknown.

Plant body sizes, on the other hand, are highly regulated by cellular activity/behavior in meristematic region located at shoot and root meristems as well as inner tissues in stems. Cell proliferation and expansion are fundamental mechanism for plant growth and development. H^+^-ATPase activity is involved in various plant physiological functions such as mineral nutrition in roots, metabolite translocation, and cellular growth (Arango et al. 2002). Relation of PATROL1 in intercellular localization of H^+^-ATPase in tissues rather than stomata has not been investigated yet.

The biomass of a local organ is not determined by the function of that organ alone, but by the balance of the entire plant. Root-to-shoot interactions are crucial for plant growth and development and many grafting practices have demonstrated such relations (Goldschmidt 2014; Tsutsui and Notaguchi 2017). Scion growth is affected by numerous aspects of rootstock, such as root hydraulic pressure, water uptake efficiency, hormone profile, and nutrient uptake (Warschefsky et al. 2016). Among grafting practices, control of shoot size and aspects by exchanging rootstocks has been demonstrated in vegetables and fruit trees. In tomatoes, seedling vigor was varied by exchanging the rootstock genotype (Hu et al. 2016). Rootstock vigor exhibited a role in the increase of shoot yield (Masterson et al. 2016). In grapevines, rootstock effects on water use efficiency are reflected in net photosynthesis, CO_2_ assimilation rate, stomatal conductance, and carboxylation efficiency, especially under water stress conditions (Iacono et al. 1998; Soar et al. 2006). In trees, vigor reduction or dwarfism has been achieved using polyploid rootstocks, a very common effect of genome duplication on trees (Ruiz et al. 2020). Thus, roots significantly affect to shoot size and architecture.

In this study, we investigated the *PATROL1* function in whole seedling using its loss-of-function mutant and overexpressing line. Grafting experiments were performed using the *patrol1* mutant, the wild-type, and an overexpressing line to examine whether the action of *PATROL1* in roots has impacts on shoot size increase. In the grafting experiments, thermal imaging analysis of the scion shoots was conducted to distinguish between the action of *PATROL1* in roots and the stomatal response. The mode of action of PATROL1 in roots was investigated by observing the plasma membrane localization of AHA1 in the roots of *patrol1* mutant.

## Materials and methods

### Plant materials and growth conditions

All Arabidopsis lines used in this study were derived from a Columbia background (Col-0). Plants were grown on 1% agar medium containing 1/2 × Murashige and Skoog (MS) salt in a growth chamber (constant white light of 80 µmol m^–2^ s^–1^ at 22 °C, 60% RH) or under conditions for grafting experiments as shown below, and, if required for further analysis, plants were transplanted into vermiculite pots. All intact and grafted plants transferring to vermiculite pots were watered with Hyponex liquid fertilizer (NPK = 6–10–5; Hyponex Japan, Osaka, Japan) diluted 1:2000.

### β-Glucuronidase (GUS) staining

Histochemical staining by GUS activity in *PATROL1* promoter*::GUS* transgenic plants was assayed with 5-bromo-4-chloro-3-indolyl-D-glucuronide as substrate (Hashimoto et al. 2006). Plant seedlings were soaked in 0.3% Triton X-100, 10 mM ethylenediaminetetraacetic acid, 1.9 mM X-Gluc, and 0.5 mM potassium ferricyanide, and incubated at 37 °C for 16 h. GUS staining was stopped by soaking in 70% ethanol and observed under a microscope.

### Micrografting

I-shaped micro-grafting of Arabidopsis was performed (Turnbull et al. 2002; Notaguchi et al. 2009), with some modifications. Briefly, a wedge-shaped graft was assembled on the hypocotyl of 4-day-old seedlings grown on 1/2 × MS medium containing 0.8% agar at 22 °C. These grafting procedures were performed on nylon membranes, and seedlings were grown for 5–6 days on 1/2 × MS medium containing 1.5% agar at 27 °C. Grafted seedlings were subsequently transferred to 1/2 × MS medium containing 0.8% agar and grown at 22 °C until used for analysis. For thermal imaging, grafted seedlings were transferred to the soil and grown for an additional seven days.

### Thermal imaging

Grafted plants were grown on a plate for two weeks and transferred to the soil for one week. They were placed in a growth cabinet (constant white light of 40 µmol m^–2^ s^–1^ at 22 °C and 43% RH) equipped with an automatic CO_2_ control unit (FR-SP; Koito, Tokyo, Japan) (Hashimoto et al. 2006). Thermal images of the plants were captured under different CO_2_ conditions using a thermography apparatus (TVS-8500; Nippon Avionics, Yokohama, Japan) (Hashimoto et al. 2006).

### Imaging with scanning *electron* microscopy

The first true leaves of 20-day-old *patrol1* mutant, wild-type, and *PATROL1*-overexpressor were sampled for scanning electron microscopy (SEM) imaging. Soon after leaf sample collection, the leaf was placed with the adaxial side up on the stage using a carbon double-tape. Then, SEM images of leaf adaxial epidermis were immediately obtained at a magnification of 500 × using a digital microscope (VHX D510; Keyence, Osaka, Japan) according to the manufacturer’s instructions.

### Immunohistochemistry

Immunohistochemical staining was carried out as described previously with minor modifications (Pasternk et al. 2015). In brief, root tips isolated from 5-day-old seedlings were fixed with methanol. After cell wall digestion and membrane permeabilization, the samples were blocked with 2% BSA in MTSB buffer. We used a specific anti-H^+^-ATPase antibody as a primary antibody, raised against the catalytic domain of AHA2 recognizing main isoforms of PM H^+^-ATPase in Arabidopsis, both AHA1 and AHA2 (Hayashi et al. 2024). As a secondary antibody, Alexa Fluor488 goat anti-rabbit IgG (Thermo Fisher Scientific, Waltham, MA, USA) at a dilution of 1: 1000 was applied. Each specimen was mounted on a glass slide with antifade mounting medium ProlongGlass (Thermo Fisher Scientific).

### Confocal microscopy

Four-day-old seedlings were incubated with propidium iodide (PI; 1 mg/mL). Samples of seedling roots were collected on a slide and covered with coverslips. Confocal laser scanning microscopy (CLSM) of the PI-stained samples was conducted using an upright FV1000 CLSM (Olympus, Tokyo, Japan). The excitation wavelength was 559 nm, and the transmission range for emission was 655–755 nm. Images were obtained using Olympus FluoView software. For estimation of AlexaFuolo488 fluorescence intensities, all images were taken at identical exposure times with a combination of a narrow excitation band-pass filter set: BP460–495 BA510–550 (U-MNIBA3; Olympus).

To observe fluorescent proteins, confocal sections were acquired using a microscope (IX-70; Olympus) equipped with a 100 × objective lens (UPlanSApo, NA = 1.40; Olympus), scientific CMOS camera (Prime 95 B; Teledyne Photometrics, Tucson, AZ, USA), and spinning disc confocal unit (CSU-X1; Yokogawa, Tokyo, Japan). Green fluorescent protein, GFP, and red fluorescent protein, RFP, were excited at 488 nm and 555 nm, with emission wavelengths of 510–550 nm and 617–673 nm, respectively, through a band-pass filter. To visualize the dynamics of GFP-PATROL1 dots, we obtained the maximum intensity projection images from the time-lapse images using the ‘Temporal-Color Code’ ImageJ plugin (https://imagej.net/plugins/temporal-color-code) (Sato et al. 2021). To examine the colocalization of GFP-PATROL1 and an endocytic marker FM4-64 (Thermo Fisher Scientific), we stained the roots with 0.8 μM FM4-64 for 2 min and then washed them with distilled water. The root cells were immediately observed. FM4-64 was excited at 488 nm, and the fluorescence was detected through a 617–673 nm band-pass filter. To evaluate the sensitivity to phenylarsine oxide (PAO), the seedlings were immersed into the distilled water containing 0.1% dimethyl sulfoxide or 20 μM PAO and then observed using the confocal microscope.

### Root acidification

Plants were germinated in 1/2 × MS medium for 10 days, transferred to that with 1 mM 2-(*N*-morpholino) ethanesulfonic acid, pH 6.8 and 0.04 g/L bromocresol purple and incubated for 24 h (Yang et al. 2007). Acidification of medium was indicated by the intense yellow color of the pH indicator dye.

### Statistical analysis

Statistical analysis was performed using Student’s* t*-test for independent samples with equal variance. A *P*-value less than 0.05 was considered statistically significant. Multiple comparisons were performed using the Tukey–Kramer HSD. Python (3.12.2), numpy (1.26.4), scipy (1.13.0), pandas (2.2.1), statsmodels (0.14.1) and scikit-posthocs (0.9.0) were used for statistical processing.

## Results

### PATROL1 affects cell morphology in leaves and meristematic activity in roots

**Fig. 1:**
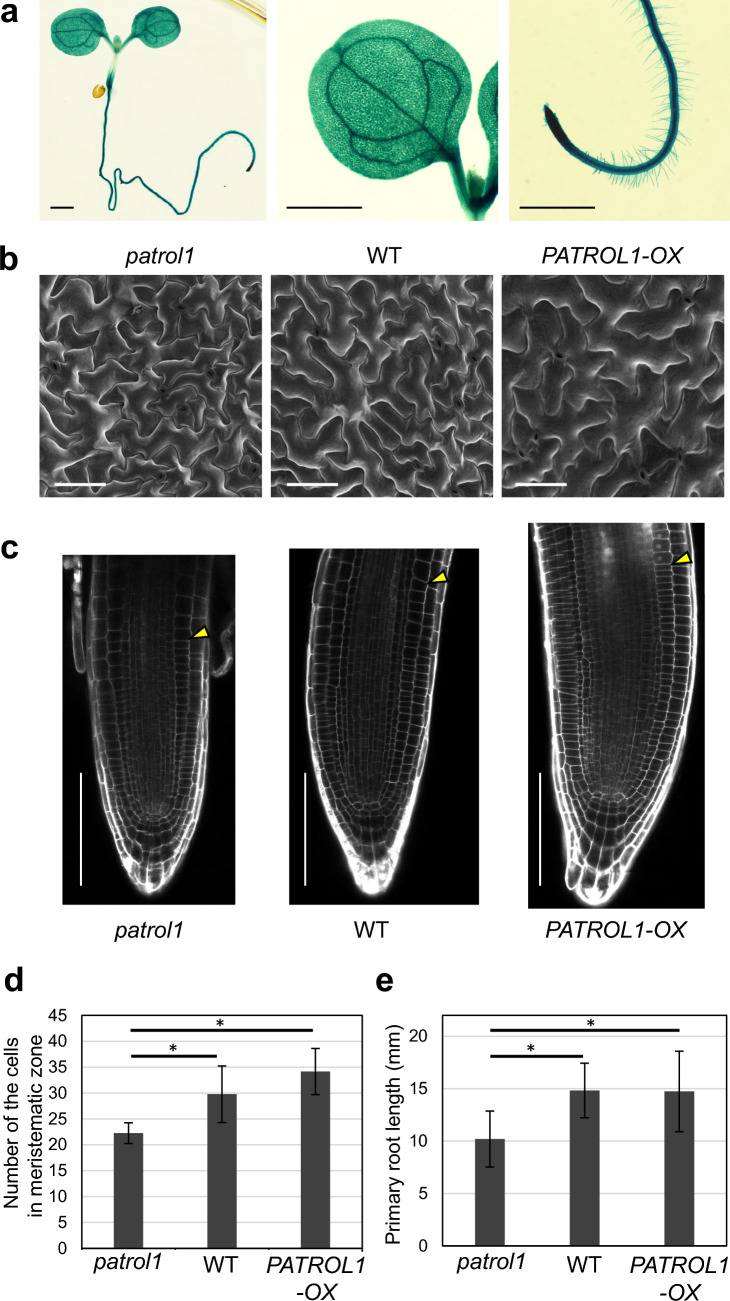
*PATROL1* is expressed in leaves and roots and affects the cell size. **a** Expression of *PATROL1* promoter*::GUS* in an 11-day-old seedling*.* GUS was strongly expressed in the whole plant (left), especially in the leaves (middle) and roots (right). Scale bars indicate 1 mm. **b** SEM images of leaf adaxial epidermis of *patrol1* mutant, wild-type (WT), and *PATROL1*-overexpressor (*PATROL1-OX*) captured by Keyence VHX D510, X500. Cells were smaller in *patrol1* and larger in *PATROL1*-OX than those in WT. Scale bars indicate 100 nm. **c** Propidium iodide (PI)-stained images of root meristems in *patrol1* mutant, WT, and *PATROL1-OX* captured by FV1000. White arrowheads indicate the junction between the meristematic and elongation zones. Scale bars indicate 100 μm. **d,e** The number of the cells in meristematic zones (**d**) and the primary root length (**e**) of the root of *patrol1* mutant, WT, and *PATROL1-OX. n* = 9 –12 (**d**) and 33–48 (**e**). Differences between the sample groups were tested using two-way ANOVA followed by a Tukey–Kramer HSD (asterisks indicate *P* < 0.01)

The expression pattern of PATROL1 was examined using the *PATROL1* promoter*::GUS* line. Strong expression of GUS was observed in whole seedlings, as reported previously (Hashimoto-Sugimoto et al. 2013, Fig. 1a). GUS was expressed ubiquitously in the leaves and roots, suggesting that PATROL1 functions in these tissues. We examined the morphology in these tissues of *patrol1* mutant, wild-type, and *PATROL1*-overexpressor. The adaxial epidermis of the first true leaf was observed using SEM. Average size of the epidermal pavement cells did not statistically differ among the *patrol1* mutant, wild-type and *PATROL1*-overexpressor. However, the shape and surface texture of the pavement cell showed different aspects. The *patrol1* mutants had more sharply angled projections, while *PATROL1*-overexpressor had fewer projections and were generally round compared to the wild-type (Fig. 1b). Next, CLSM was used to observe the root cytological analysis of *patrol1* mutant and overexpressor. The size of cells in the meristematic zone was not statistically different among the *patrol1* mutant, wild type and PATROL1-overexpressor (Suppl. Fig. S1). The meristematic zone, however, in the *patrol1* mutant was smaller than in the wild-type, and the *PATROL1*-overexpressor had a larger meristematic zone than the *patrol1* mutant and wild-type (Fig. 1c). The number of cells in the meristematic zone of *patrol1* mutant was significantly less than those of the wild-type. Conversely, the number of cells in *PATROL1*-overexpressor was not significantly but slightly higher than those in the wild-type (Fig. 1d). Consistent with the result, the primary root lengths of *patrol1* mutant and *PATROL1*-overexpressor were shorter and longer than those of the wild-type, respectively (Fig. 1e).

### Functions of endogenous *PATROL1* in roots contributes to the shoot size of scion in grafts

**Fig. 2:**
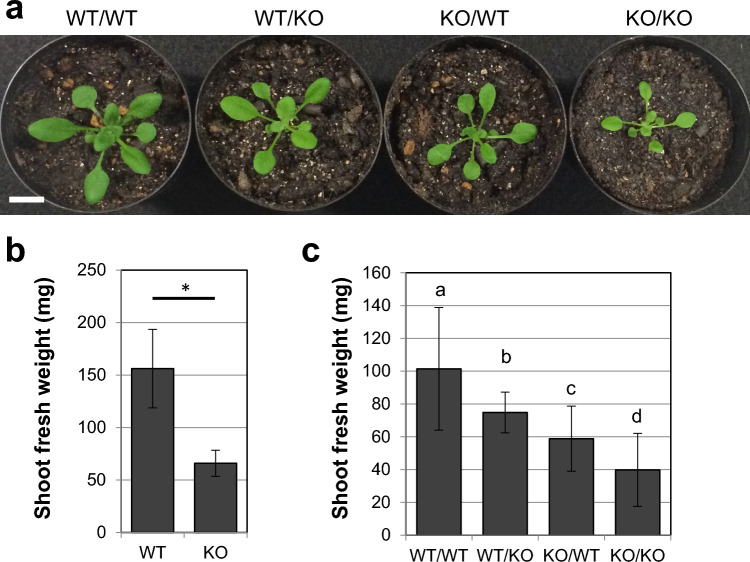
Shoot and root growth in grafts of wild-type and the *patrol1* mutant. **a** Reciprocal micrografting using wild-type (WT) and *patrol1* knockout mutant (KO) was performed. Images of grafted plants at 14 days after grafting are shown. Scale bar indicates 2 cm. **b,c** Shoot fresh weight was measured for intact WT and KO (**b**) and each graft combination (**c**) (*n* = 10–18). An asterisk indicates statistical significance (Student’s t-test, *P* < 0.05). Differences between the sample groups were tested using two-way ANOVA followed by a Tukey–Kramer HSD (between **b** and **c**, *P* < 0.05; other combinations, *P* < 0.01)

To further investigate the PATROL1 function in the shoot and root, we performed reciprocal grafting of the wild-type and *patrol1* mutant. I-shaped shoot–root grafts were assembled on the hypocotyl of 4-day-old seedlings, and the shoot of the scion plants appeared to be successfully grafted 14 days after grafting in the soil pots (Fig. 2a). The shoot of the wild-type self-graft was larger than that of the *patrol1* mutant self-grafts. Wild-type shoots grafted onto a *patrol1* mutant rootstock and a *patrol1* mutant grafted onto a wild-type rootstock exhibited intermediate sizes. We measured the shoot fresh weight of the four graft combinations together with the same-aged intact seedlings of wild-type and *patrol1* mutants, which were grown in the same manner as grafts without grafting surgery (Fig. 2b). The shoot fresh weight of the intact wild-type plants was significantly higher than that of the intact *patrol1* mutant plants (Fig. 2b). The size of *patrol1* mutant scions grafted onto a wild-type rootstock was significantly larger than the ones grafted onto a *patrol1* mutant rootstock (Fig. 2c), suggesting that the function of the endogenous PATROL1 in roots contributed to the increase in shoot size. In contrast, grafting of the *patrol1* mutant rootstock significantly decreased the fresh weight of wild-type shoots (Fig. 2c). This result confirmed that the function of endogenous PATROL1 in the roots contributes to the increase in shoot size.

### Overexpression of *PATROL1* in roots increases shoot biomass of scion in grafts

**Fig. 3:**
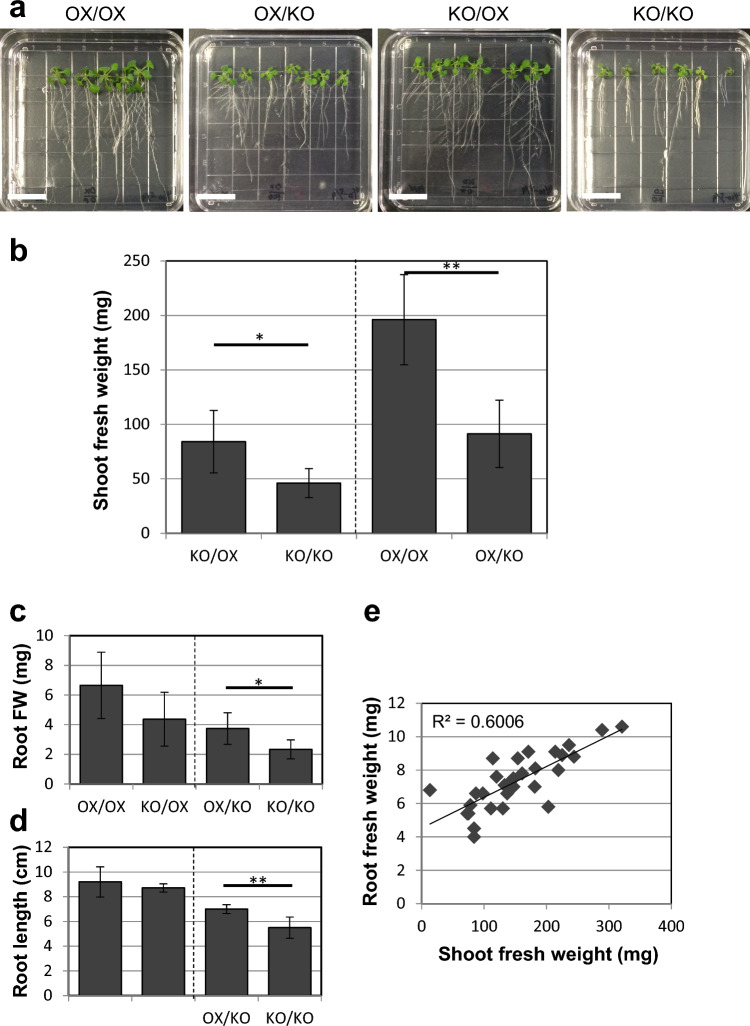
Shoot and root growth in grafts of *PATROL1* overexpressor and *patrol1* mutant. **a** Reciprocal micrografting using *PATROL1* overexpressor (OX) and *patrol1* knockout mutant (KO) was performed. Images of grafted plants 14 days after grafting are shown. Both shoot and root sizes were apparently different between OX/OX and KO/KO self-grafts, being large and small, respectively. The shoot and root size of the reciprocal graft combinations, OX/KO and KO/OX, were intermediate compared to those of the self-grafts. Scale bars indicate 2 cm. **b** Shoot fresh weight was measured for OX (left) and KO (right) grafted on OX and KO (*n* = 5–12). **c–e** Another set of micrografting experiments showing the root fresh weight (**c**, root FW, mg) and the root length (**d**, cm) (*n* = 6). There is a positive correlation between root and shoot fresh weight (**e**). Student's t-test was performed for the comparable combinations with the same genotype of shoot (**b**) or root (**c**, **d**) and each combination was separated by a dashed line. Asterisks indicate statistical significance (*, *P* < 0.05; **, *P* < 0.01)

We further confirmed that *PATROL1* function in roots can increase shoot growth using a *PATROL1*-overexpressor. We performed grafting experiments using *PATROL1*-overexpressor and *patrol1* mutants (Fig. 3). The seedlings were grown on agar plates 14 days after grafting (Fig. 3a). *PATROL1*-overexpressor self-grafts exhibited a larger quantity of shoots and roots than the *patrol1* mutant self-grafts. The shoots of *patrol1* mutant scions grafted onto the *PATROL1*-overexpressor rootstock were obviously larger than those of *patrol1* mutant self-grafts. Moreover, *patrol1* mutant rootstocks grafted with *PATROL1*-overexpressor scion grew more roots than the *patrol1* mutant self-grafts.

We measured the shoot fresh weight of these four graft combinations 14 days after grafting (Fig. 3b). The *patrol1* mutant shoot weight was significantly increased by grafting onto the *PATROL1*-overexpressor rootstock compared with that of *patrol1* mutant self-grafts (Fig. 3b). This suggests that overexpression of *PATROL1* in rootstocks can increase the shoot biomass of scions in grafts. In contrast, grafting of the *patrol1* mutant rootstock drastically decreased the shoot size of the *PATROL1*-overexpressor scion compared to the case of *PATROL1*-overexpressor self-grafts (Fig. 3b). This result again confirmed that the function of the *PATROL1* in roots is important for the increase in shoot biomass.

### Overexpression of *PATROL1* in shoots increases growth of rootstock in grafts

Since roots contribute to the shoot growth and shoots drive root growth, we examined whether *PATROL1* function in shoots can contribute to root growth. Root growth in reciprocal grafts of the *PATROL1*-overexpressor and *patrol1* mutant was quantified by measuring the root fresh weight (Fig. 3c) and primary root length (Fig. 3d). Significant differences were detected both in the root fresh weight and in the primary root lengths of the *patrol1* mutant rootstocks grafted with *PATROL1*-overexpressor and *patrol1* mutant. In the case of grafts where *PATROL1*-overexpressor and *patrol1* mutant were grafted onto *PATROL1*-overexpressor rootstock, no significant difference was detected in the *PATROL1*-overexpressor rootstock. These findings suggest that the shoot *PATROL1* function is exhibited more when the PATROL1 function is absent in the roots. By collecting data of these four graft combinations, a positive correlation between root and shoot fresh weights was identified (R^2^ = 0.74), suggesting that shoot size reflects root biomass and, vice versa, root size reflects shoot biomass (Fig. 3e). Thus, it is indicated that not only does *PATROL1* overexpression in roots increase shoot biomass, but *PATROL1* overexpression in shoots can also increase root biomass through grafting.

### Stomatal movement controlled by *PATROL1* is driven in cell-autonomous manner

**Fig. 4:**
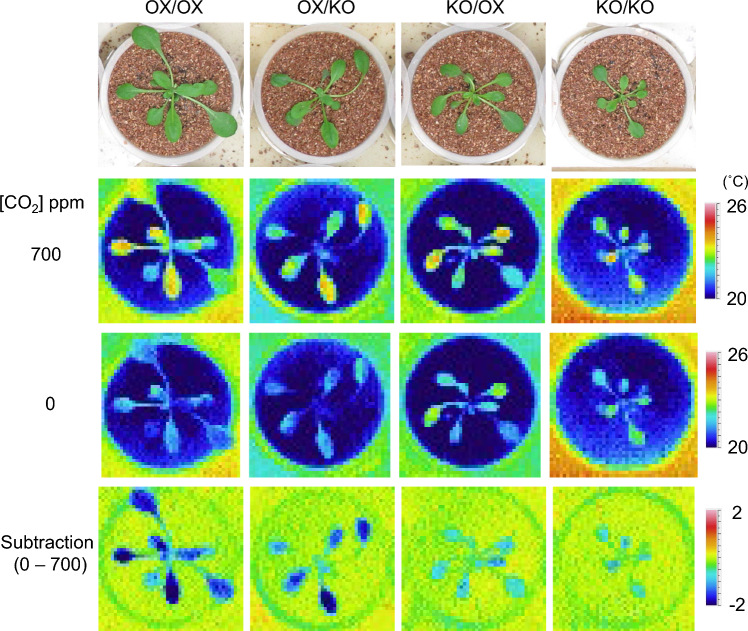
CO_2_ response indicated by leaf temperature of grafted plants consisting of *PATROL1* overexpressor and *patrol1* mutant. Grafted plants grown on plate for 14 days and in soil for 7 days were subjected to the indicated CO_2_. The subtractive images show changes in leaf temperature in response to change from high to low CO_2_

We found that root and shoot PATROL1 each affect mutual biomass. To test the possibility that PATROL1 function in roots affects stomatal opening by grafting, we examined stomatal CO_2_ response monitored by thermography in the reciprocal grafts of the *PATROL1*-overexpressor and *patrol1* mutant. Under low CO_2_ condition, stomatal opening and enhanced transpiration are induced, resulting in lower leaf temperature in PATROL1-overexpressor plants. In contrast, leaf temperature remains high in *patrol1* because of a defect in stomatal opening in response to low CO_2_ (Hashimoto-Sugimoto et al. 2013). Grafts were transplanted into the soil 14 days after grafting and grown for additional 7 days. Thermal images of the plants were captured under different CO_2_ conditions (0 and 700 ppm) using a thermography apparatus (Hashimoto et al. 2006) 21 days after grafting (Fig. 4). The subtractive images (0 ppm–700 ppm) show changes in leaf temperature when the grafted plants were transferred from high to low CO_2_ conditions. The leaf temperatures of the grafted plants were consistent with their respective shoot genotypes regardless of their rootstocks, indicating that stomatal movement was under cell-autonomous regulation. Thus the *patrol1* mutant shoot grafted onto the *PATROL1*-overexpressor could increase the biomass with no change in stomatal responsiveness. Therefore, the cause of the increase in shoot growth by rootstock of PATROL1 was independent of the effect of stomatal aperture.

### Unidentified regulatory target of PATROL1 in roots

**Fig. 5:**
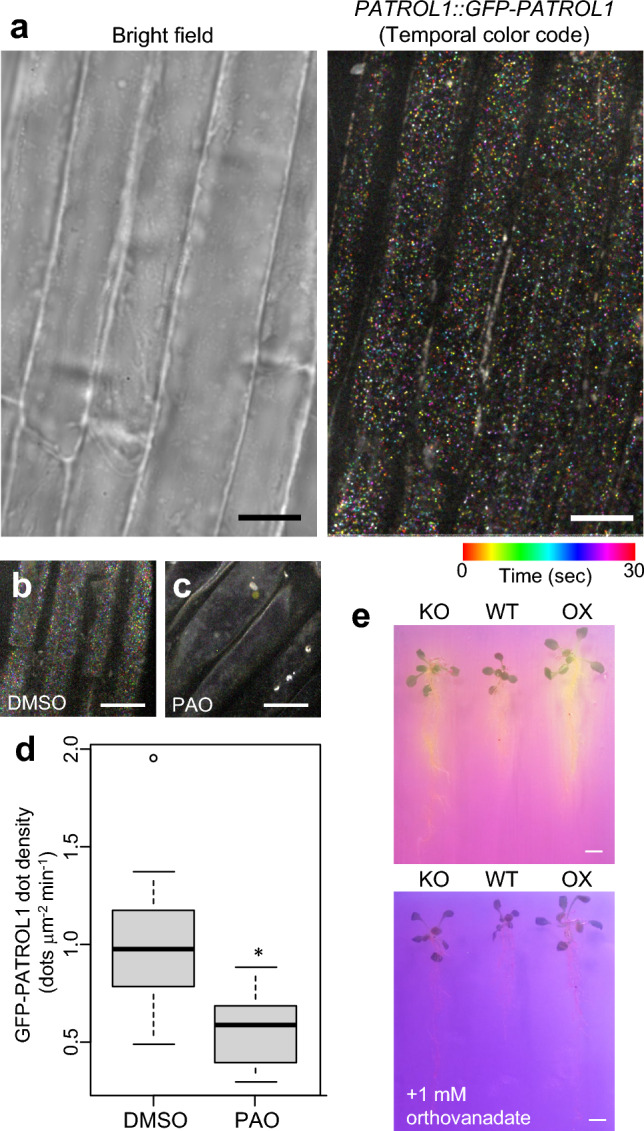
Dynamics of the GFP-PATROL1 dots in the root cells. **a** Representative bright field image of the root cells and time-projection image of GFP-PATROL1 in the cells. The time-sequential images were acquired at 500 ms intervals for 30 s (60 frames) and projected using the ImageJ ‘Temporal-Color Code’ function. The scale bar indicates 10 mm. **b, c** Effects of PI4K inhibitor PAO treatment on GFP-PATROL1 dots. Representative time-projection image of GFP-PATROL1 in root cells treated without (**b**) or with 20 μM PAO (**c**). The scale bar indicates 10 μm. **d** Density of GFP-PATROL1 dots (*n* = 35). Significance was determined by Mann–Whitney test. **e** Assessment of acid release from roots using bromocresol purple, a pH indicator, with or without orthovanadate treatment. Images were taken one day after transferring 10-day-old seedlings of *patrol1* mutant (KO), wild-type (WT), and *PATROL1*-overexpresssor (OX) to the medium containing bromocresol purple. The scale bar indicates 1 cm

In guard and subsidiary cells, GFP-tagged PATROL1 localizes on the small dots just beneath the plasma membranes in phosphoinositide 4-kinase (PI4K)-dependent manner, and the GFP-PATROL1-labeled dots resides for several seconds (Hashimoto-Sugimoto et al. 2013; Higaki et al. 2014). These GFP-PATROL1 localizations and dynamics are suggested to be closely related to its function in plasma membrane delivery of H^+^-ATPases. To examine whether PATROL1 contributes to similar membrane trafficking to the plasma membrane in root cells, we observed the intracellular localization and dynamics of PATROL1 in the root cells using a GFP-PATROL1 transgenic line generated previously (Hashimoto-Sugimoto et al. 2013). As in the case of guard and subsidiary cells, GFP-PATROL1-labeled small dot patterns were observed in the root cells (Fig. 5a). The small dots appearing on the plasma membranes then disappeared after residing at the same site for several seconds (Supplementary Movie S1), as previously observed in the stomatal guard and subsidiary cells. In addition, the root cell GFP-PATROL1 dots were sensitive to the inhibitor of PI4K, phenylarsine oxide (PAO) (Fig. 5b–d), as in the case of leaf cells (Higaki et al. 2014). These results suggest that PATROL1 contributes to plasma membrane delivery of target proteins in a PI4K-dependent manner, both in leaf and root cells.

H^+^-ATPases could be a target protein of PATROL1 in root cells, like in stomatal cells, and action of plasma membrane H^+^-ATPases could enhance proton exports to outside of root cells. Since increase of protons causes acidic conditions, we examined acid release from the roots in *patrol1* mutant, wild-type, and *PATROL1*-overexpresssor. Acid release from roots was visualized by transferring 10-day-old seedlings to a gel containing bromocresol purple, a pH indicator, and treated for one day. Acid release was observed in all genotypes. Compared to wild-type, the *patrol1* mutant showed slightly less acid release while *PATROL1*-overexpresssor showed greater acid release (Fig. 5e, upper panel). As a control experiment 1 mM of orthovanadate, a H^+^-ATPase inhibitor, was treated, and the roots were observed. The orthovanadate-treated roots showed almost no acid release (Fig. 5e, lower panel). These results may indicate that plasma membrane H^+^-ATPases are the target of PATROL1 in roots. We then examined whether AHA1, the target of PATROL1 in stomatal cells, is a target of PATROL1 in roots. We first confirmed that the RFP-AHA1 signal was observed in the plasma membrane in stomatal guard cells of cotyledons and that the plasma membrane localization pattern of RFP-AHA1 was abolished in the *patrol1* mutant in stomatal guard cells of cotyledons (Suppl. Fig. S2a, Hashimoto-Sugimoto et al. 2013). We next investigated whether *PATROL1* also regulates the localization of AHA1 in root cells. We checked RFP-AHA1 localization in root cortex cells, and it was found to be localized near the cell surface and mid-plane in the wild-type and *patrol1* mutant backgrounds (Suppl. Fig. S2b). Different from guard cells, the RFP-AHA1 signal in root cells was strong in both wild-type and *patrol1* mutant, and no difference in the RFP-AHA1 localization pattern was found, implying that the regulatory target(s) of PATROL1 other than AHA1 in root cells are responsible for the increase in organ size. AHA1 and AHA2 are the predominant plasma membrane H^+^-ATPases (Harper et al. 1990). AHA1 is found all over the plant, whereas AHA2 plays its major role in roots and positively regulates primary root growth, possibly by promoting cell expansion (Hoffmann et al. 2019). We conducted immunostaining analysis for estimating the H^+^-ATPase amount on the plasma membrane not only of AHA1 but also AHA2, using anti-H^+^-ATPase antibody raised against the catalytic domain of AHA2, similarly recognizing both AHA1 and AHA2 (Hayashi et al. 2024). Plasma membrane-localized H^+^-ATPase amount estimated from fluorescence intensity was not significantly different between wild-type and the *patrol1* mutant (Suppl. Fig. S3). The amount of AHA1 and AHA2 on the plasma membrane do not seem to explain why the roots are shorter in the *patrol1* mutant.

## Discussion

PATROL1 is involved in the regulation of stomatal movement, which determines the degree of carbon fixation and consequently affects plant body size. Plasma membrane H^+^-ATPase AHA1 is a target molecule involved in PATROL1-mediated intercellular transport to the plasma membrane, because its abundance at the plasma membrane depends on PATROL1 (Hashimoto-Sugimoto et al. 2013; Higaki et al. 2014). Previous studies demonstrated that overexpression of H^+^-ATPase in stomatal guard cells using the GC1 guard cell-specific promoter or in the entire tissues using the 35S promoter showed an increase in plant size through increased photosynthetic rate as a result of enhanced stomatal opening; the GC1 promoter drives AHA2 in Arabidopsis (Wang et al. 2014), the GC1 promoter drives AHA2 in poplar (Zhang et al. 2021). These studies have demonstrated the importance of stomatal control in carbon fixation and plant growth. PATROL1 is not only expressed in stomatal guard cells but ubiquitously expressed in shoot and root tissues, raising the possibility that PATROL1 regulates organ size beyond its previously reported role in controlling stomatal movement (Hashimoto-Sugimoto et al. 2013). In grafting experiments, root PATROL1 was found to affect shoot biomass (Figs. 2 and 3). Leaf temperature of the grafted plants measured by thermography showed that root PATROL1 did not affect shoot stomatal aperture (Fig. 4). Furthermore, overexpression of *PATROL1* was shown to increase shoot and root biomass by grafting. These results indicate that PATROL1 plays a role in regulating organ size throughout the seedling by a mechanism other than stomatal control (Figs. 2–4).

Cytological observations of the *patrol1* mutant and *PATROL1*-overexpressor in the leaf epidermis and root tip cells indicate that PATROL1 affects size at the organ level by affecting cell complexity and cell proliferation activity (Fig. 1). We have demonstrated a role of PATROL1 in roots that affects root meristem size. As demonstrated in stomatal cells, AHA1 is potential target molecule involved in PATROL1-mediated phenomena because its abundance at the plasma membrane depends on PATROL1. Moreover, PATROL1 protein localization patterns in the root cortex showed conserved behavior of the MUN domain protein (Fig. 5, Supplementary Movie S1). Although this hypothesis appeared to be correct because the *patrol1* mutant roots released less acid and the overexpressors released more acid (Fig. 5e), it was revealed that the amount of AHA1 and AHA2 present in the root epidermis was not less in the *patrol1* mutants (Suppl. Figs. S2 and S3). Root meristematic size is determined as the initial steps of cell differentiation and depend on the concomitant activation by the plant hormone cytokinin and H^+^-ATPases (Elena et al. 2018). Plasma membrane H^+^-ATPases are found throughout the plant in every cell type examined, but certain cell types have much higher concentrations of H^+^-ATPases than others (Ueno et al. 2005). *PATROL1* expressed strongly in vascular tissues and various cell types, may influence cell growth by localizing H^+^-ATPases other than AHA1 and AHA2 to the plasma membrane. Root expressed genes encoding H^+^-ATPases such as AHA3, AHA4, AHA7 and AHA8 may be potential targets (Ueno et al. 2005).

Suppression of some H^+^-ATPases expressed in the phloem induces retarded growth, probably because the inability to load photosynthetic assimilates into the phloem (Zhao et al. 2000). Some H^+^-ATPases expressed in root endodermis are considered to have a role for the pump in the active loading of solutes into the xylem (Parets-Soler et al. 1990). Protons in the apoplast are thought to activate pH sensitive enzymes and proteins within the cell walls, and to initiate cell-wall loosening and extension growth, known as the acid growth hypothesis (Hager 2003; Barbez et al. 2017; Li et al. 2021). Thus, H^+^-ATPases can contribute to increasing plant biomass in various aspects. On the other hand, it has been reported that PATROL1 is involved in the delivery of cellulose synthase complexes to the plasma membrane (Zhu et al. 2018). It is possible that PATROL1 targets different molecules to control their cellular localization rather than H^+^-ATPases in the roots. Identification of the target molecules of PATROL1 in tissues other than the stomatal and subsidiary cells is a future task.

Grafting experiments revealed the importance of *PATROL1* function in increasing the shoot and root size (Figs. 2 and 3). Furthermore, a correlation between shoot and root biomass indicated the importance of both shoots and roots for the growth of the rest of the plant (Fig. 3e). Thermal imaging analysis demonstrated that the expression level of *PATROL1* in roots was found to be independent of stomatal response but affected shoot biomass, demonstrating that *PATROL1* is involved in size control without stomatal involvement (Fig. 4). Furthermore, we demonstrated the usefulness of the *PATROL1*-overexpressor as a grafting plant to enhance both the shoot and root biomass of the graft partner (Fig. 3). Grafting experiments using different shoot and root plant genotypes have also been previously reported. For example, in grafted grapevines, a positive correlation between shoot and root biomass was identified in one graft combination but not in the other (Tandonnet et al. 2010). Meanwhile, an inconsistent effect of the rootstock was also observed. It has been argued that rootstocks can contribute to the shoot performance, such as gas exchange and other photosynthetic parameters, but the extent of the rootstock effect depends on environmental conditions. The intrinsic water-use efficiency was different when rootstocks were exchanged, and variations in photosynthetic parameters were only found under drought or salt stress conditions and not under non-stress conditions (Fullana-Pericàs et al. 2020). In addition to photosynthetic performance, other plant physiological aspects were also modified on exchanging the rootstock. The amount of abscisic acid uptake or generation in the rootstock appears to be related to the stress tolerance behavior of the shoot, including stomatal closure and chilling tolerance (Iacono et al. 1998; Soar et al. 2006; Liu et al. 2016; Lv et al. 2022). The salt tolerance of grafts was achieved through the abscisic acid function on upregulation of the Ca^2+^-storage protein calreticulin in the root (Shaterian et al. 2004). Phosphorus uptake and translocation by rootstocks also influence scion shoot growth (Grant and Matthews 1998). Thus, it would be interesting to investigate how *PATROL1* function in roots affects the physiological aspects of shoots. Various stress-tolerance characteristics are crucial for achieving plant vigor under fluctuating natural conditions. The advantages of some rootstocks in gaining stress tolerance have been previously explained (Schwarz et al. 2010). *PATROL1* is a potential target for designing the proper size of plants while maintaining plant responses to changing environments. Recent studies have used genome editing methods, which is a practical way to generate new plant lines exhibiting various expression levels of endogenous genes (Rodríguez-Leal et al. 2017; Honma et al. 2020; Akagi et al. 2022). *PATROL1* orthologs are found in many crops including sorghum, grape, and rice (Hashimoto-Sugimoto et al. 2013). Thus, the results of the present study provide an important insight for gaining crop yields for future food security.

## Supplementary Information

Below is the link to the electronic supplementary material.

Supplementary file1 Movie S1 Dynamic motion of GFP-PATROL1 dots in the root cells. Time-sequential images were acquired at 500 ms intervals for 30 s (60 frames). (AVI 6099 KB)

Supplementary file2 Fig. S1 The height of the cells in the meristematic zone of the root of the patrol1 mutant, wild-type (WT), and PATROL1-OX. The vertical length of cortical cells secondary from outer layer were measured for five independent plants. Fig. S2 PATROL1 is involved in AHA1 localization at the plasma membrane of stomatal guard cells but not in roots. a Representative confocal images of RFP-AHA1 in guard cells of cotyledons in wild-type (WT) background (left) and patrol1 mutant (right). b Confocal images of the surface and mid-plane of the root cortex in wild-type background (left) and patrol1 mutant (right). Scale bars are 5 μm in (a) and 10 μm in (b). Fig. S3 Immunohistochemical estimation of plasma membrane H+-ATPase in 5-day-old Arabidopsis roots. Plasma membrane H+-ATPase proteins were detected using unti-Thr881 of AHA2 antibody. a Representative confocal images of the surface of the root in wild-type (WT, left) and patrol1 mutant (right). Scale bars are 10 μm. b Box plot of the fluorescence intensity of the plasma membrane of the roots in wild-type (WT) and patrol1 mutant. Crosses inside boxes show average values, horizontal lines within boxes mark the median value. Each boxplot consists of average values of roots from 11 plants. Each root average value derives from 8 to 27 cells. Significance between wild-type and patrol1 mutant was calculated using two-paired Student's t-test: P = 0.088. (PDF 811 KB)

## Data Availability

All data supporting the findings of this study are available within the paper and its Supplementary Information.

## Abbreviations

*PAO*: : Phenylarsine oxide
*patrol1*: : *proton ATPase translocation control 1*
*SEM*: : Scanning electron microscopy
