# Supplementary material for: The PATROL1 function in roots contributes to the increase in shoot biomass
Source: Planta. 2024 Sep 26;260(5):105. doi: 10.1007/s00425-024-04526-8 (PMC11427605; doi:10.1007/s00425-024-04526-8)
Supplement: Supplementary file 2 — Supplementary file2 Fig. S1 The height of the cells in the meristematic zone of the root of the patrol1 mutant, wild-type (WT), and PATROL1-OX. The vertical length of cortical cells secondary from outer layer were measured for five independent plants. Fig. S2 PATROL1 is involved in AHA1 localization at the plasma membrane of stomatal guard cells but not in roots. a Representative confocal images of RFP-AHA1 in guard cells of cotyledons in wild-type (WT) background (left) and patrol1 mutant (right). b Confocal images of the surface and mid-plane of the root cortex in wild-type background (left) and patrol1 mutant (right). Scale bars are 5 μm in (a) and 10 μm in (b). Fig. S3 Immunohistochemical estimation of plasma membrane H+-ATPase in 5-day-old Arabidopsis roots. Plasma membrane H+-ATPase proteins were detected using unti-Thr881 of AHA2 antibody. a Representative confocal images of the surface of the root in wild-type (WT, left) and patrol1 mutant (right). Scale bars are 10 μm. b Box plot of the fluorescence intensity of the plasma membrane of the roots in wild-type (WT) and patrol1 mutant. Crosses inside boxes show average values, horizontal lines within boxes mark the median value. Each boxplot consists of average values of roots from 11 plants. Each root average value derives from 8 to 27 cells. Significance between wild-type and patrol1 mutant was calculated using two-paired Student's t-test: P = 0.088. (PDF 811 KB) [file 425_2024_4526_MOESM2_ESM.pdf]

Supplementary Information

The PATROL1 function in roots contributes to the increase in shoot biomass

Michitaka Notaguchi, Manami Ichita, Takaya Kawasoe, Keina Monda, Ken-ichi Kurotani, Takumi Higaki, Koh Iba, Mimi Hashimoto-Sugimoto

- **Supplementary Figs. S1–S3.**
- **Supplementary Movie S1.**

  Dynamic motion of GFP-PATROL1 dots in the root cells. Time-sequential images were acquired at 500 ms intervals for 30 s (60 frames).

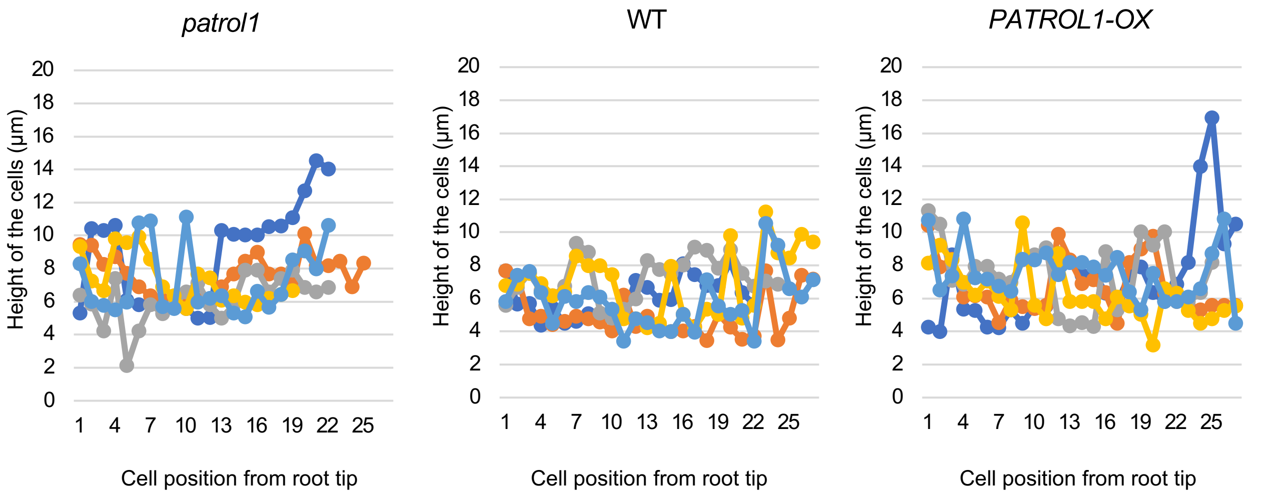

Supplementary Fig. S1. The height of the cells in meristematic zone of the root of *patrol1* mutant, wild-type (WT), and *PATROL1-OX*. The vertical length of cortical cells secondary from outer layer were measured for five independent plants.

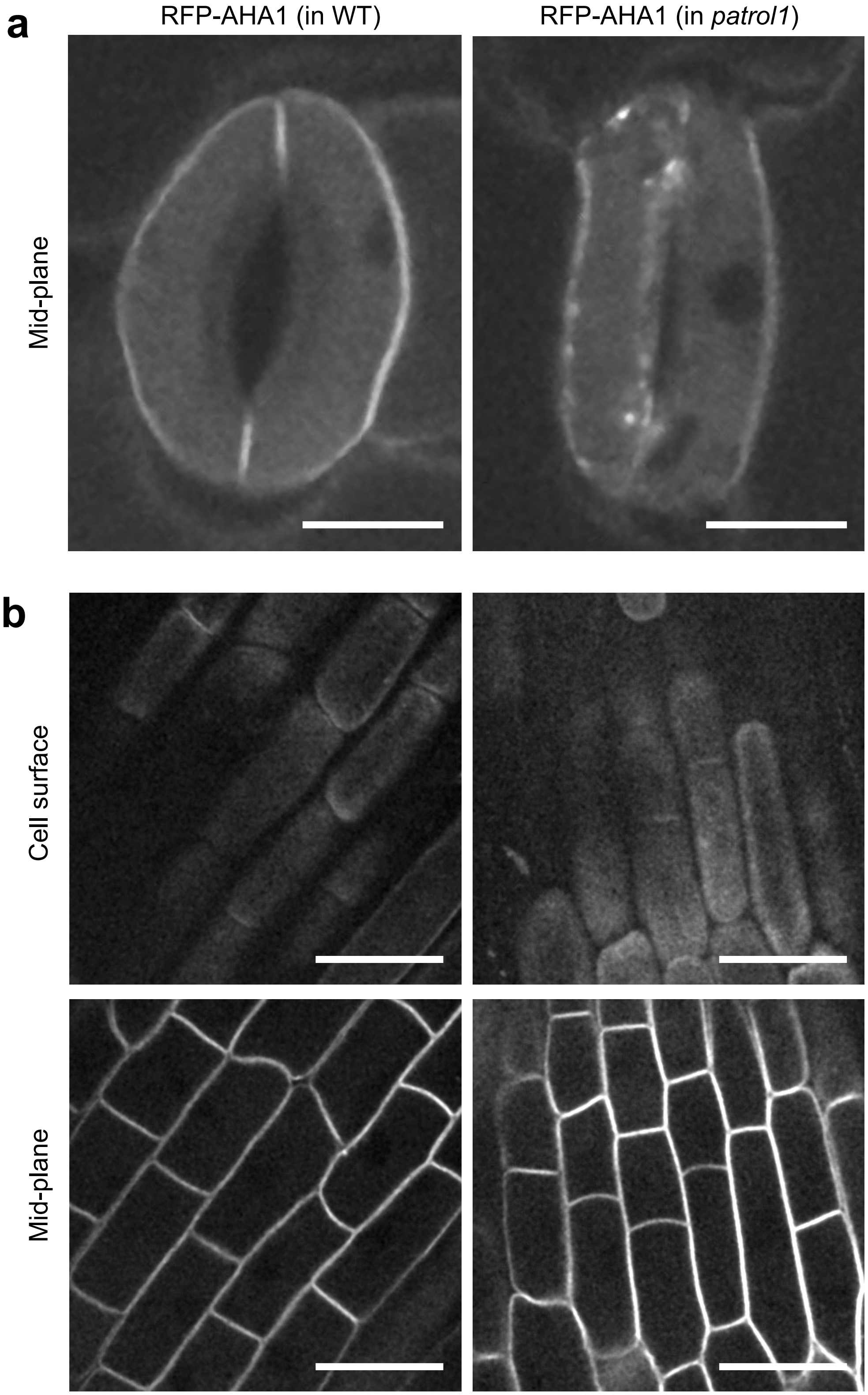

Supplementary Fig. S2. PATROL1 is involved in AHA1 localization at the plasma membrane of stomatal guard cells but not in roots. **a** Representative confocal images of RFP-AHA1 in guard cells of cotyledons in wild-type (WT) background (left) and *patrol1* mutant (right). **b** Confocal images of the surface and mid-plane of the root cortex in wild-type (WT) background (left) and *patrol1* mutant (right). Scale bars are 5 µm in (**a**) and 10 µm in (**b**).

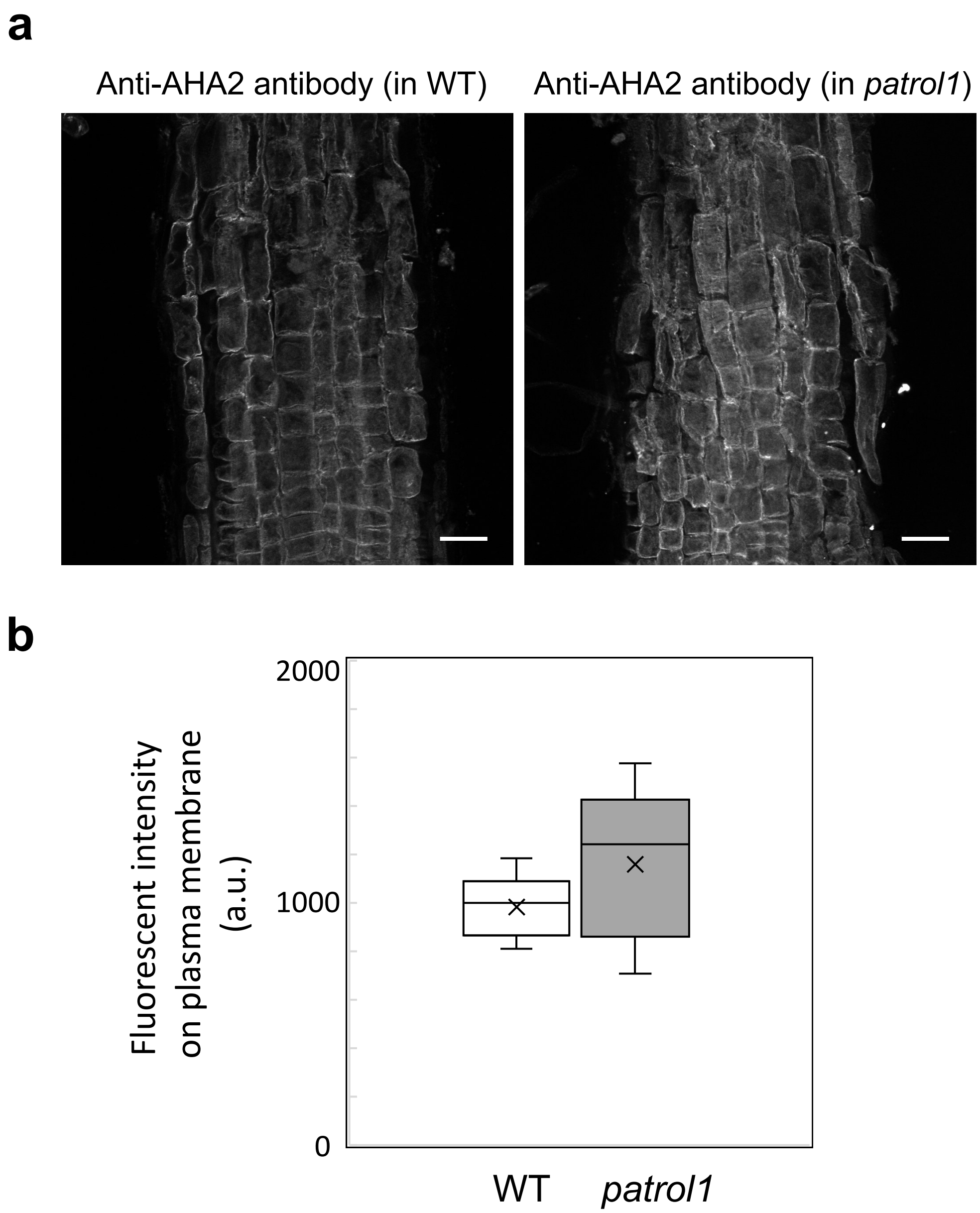

Supplementary Fig. S3. Immunohistochemical estimation of plasma membrane H^+-ATPase in 5-day-old Arabidopsis roots. Plasma membrane H^+-ATPase proteins were detected using unti-Thr881 of AHA2 antibody. **a** Representative confocal images of the surface of the root in wild-type (WT, left) and *patrol1* mutant (right). Scale bars are 10 µm. **b** Box plot of the fluorescence intensity of the plasma membrane of the roots in wild-type (WT) and *patrol1* mutant. Crosses inside boxes show average values, horizontal lines within boxes mark the median value. Each boxplot consists of average values of roots from 11 plants. Each root average value derives from 8 to 27 cells. Significance between wild-type and *patrol1* mutant was calculated using two-paired Student's t-test: $P = 0.088$.
